# Challenging Aggressive Behaviors Experienced by Personal Support Workers in Comparison to Home Care Workers: Relationships between Caregiver Experiences and Psychological Health

**DOI:** 10.3390/ijerph17155486

**Published:** 2020-07-29

**Authors:** Kelsey N. Womack, Teala W. Alvord, Caitlyn F. Trullinger-Dwyer, Sean P. M. Rice, Ryan Olson

**Affiliations:** 1 Oregon Institute of Occupational Health Sciences, Oregon Health & Science University (OHSU), Portland, OR 97239, USA; kelsnparker@gmail.com (K.N.W.); alvord@ohsu.edu (T.W.A.); trullinc@ohsu.edu (C.F.T.-D.); ricese@ohsu.edu (S.P.M.R.); 2 School of Public Health, OHSU-Portland State University (PSU), Portland, OR 97201, USA; 3 Department of Psychology, OHSU-Portland State University (PSU), Portland, OR 97201, USA

**Keywords:** challenging behaviors, workplace aggression, workplace violence, personal support workers, home care workers, caregivers, occupational safety, occupational health

## Abstract

Personal support workers (PSW) are caregivers for children and adults with intellectual and developmental disabilities (IDDs) or adults experiencing mental illness or other behavioral health conditions. The work can be very meaningful, but many PSWs must prepare for, monitor, and manage challenging behaviors, including aggression. This study was designed to estimate the prevalence of aggression experienced by PSWs in Oregon, and compare it to a previous sample of Oregon home care workers (HCWs). This comparison included an analysis of relationships between exposures to aggression and psychological health factors. PSWs in Oregon (*N* = 240) were surveyed electronically at a single time point. PSWs generally reported higher rates of exposure to aggression compared to HCWs. Experiences with aggression were positively associated with fatigue and weakly associated with depression, but not stress. PSWs’ self-reported lost work time injury rate was elevated compared to the US average, but it was comparable to previous self-reported injury rates from HCWs. Physical demands of work were the most prevalent reported primary safety concern, followed by challenging behaviors (including aggression). Developing tailored training to help PSWs understand, plan for, minimize, and manage challenging behaviors is a social priority.

## 1. Introduction

Providing in-home or community-based care for people in need is a meaningful and socially important occupation. These caregivers provide instrumental support for individuals who need assistance with basic (e.g., grooming, dressing, and toileting), instrumental (e.g., shopping, cooking, managing finances, and medications), and advanced (e.g., use of technology and volunteer work) activities of daily living (ADLs) so they can remain in their homes and neighborhoods and sustain a degree of self-governance and determination [1,2]. This care also helps individuals avoid premature transitions to more costly care environments, such as group homes or long-term care facilities. Nationally, there are approximately 3.3 million paid in-home caregivers, and this number is projected to increase by 36% between 2018 and 2028; a growth rate considerably higher than the 5% average growth for all US occupations [3]. There are varying titles for workers who provide these services (e.g., home care aids, home care workers, personal support workers, and direct care workers) and there are distinctive populations who receive different, but overlapping, types of care services. In the state of Oregon, where our study was conducted, publicly funded caregivers who provide services for adults and children with intellectual and developmental disabilities (IDDs), or adults experiencing mental illness or behavioral health conditions, are referred to as personal support workers (PSWs). Caregivers who provide services to seniors and adults with physical disabilities within publicly funded programs are called home care workers (HCWs) [4]. There are currently over 14,500 PSWs and over 18,000 HCWs working in Oregon caring for individuals who qualify for publicly funded services [5].

Although caregiving is a meaningful role, and those who are engaged in this type of paid work tend to regard it positively [6,7], it can be both emotionally and physically demanding. Indeed, qualitative accounts of PSW work experiences have illustrated feelings of compassion and meaningfulness, but also stress resulting from work conditions and challenging behaviors exhibited by consumers [8]. Previous research has shown that both HCWs [9,10] and PSWs [11] are at risk for experiencing exposure to challenging behaviors, including inter-personal aggression and violence, in the course of doing their jobs. Our study was planned to better understand the similarities and differences between PSWs and HCWs in their experiences with aggression and violence at work. Secondary goals included describing PSW demographics, and to evaluate relationships between exposures to aggression and violence and PSWs’ psychological health.

Those receiving care from PSWs and HCWs are identified by, or may prefer, different labels. For HCWs participating in Oregon’s publicly funded system, service recipients are referred to as consumer-employers. This moniker is not a perfect fit for the recipients of care from PSWs. Therefore, for the purposes of this paper, we will use the term “consumer” to identify those receiving care from either PSWs or HCWs. We also note that the words aggression and violence can suggest a level of intentionality or directed harm that may or may not be present in consumer–caregiving interactions. We will define aggression and violence for the purposes of our study in a subsequent section, but at the outset of this paper, we want to state that consumers who behave aggressively, and/or physically harm their caregivers, may not have any intention to cause emotional or physical harm.

### 1.1. Profile of Work Roles of PSWs and HCWs

PSWs assist children and adults with intellectual and developmental disabilities (IDDs), which are usually present at birth and affect the person’s ability for typical physical, intellectual, and/or emotional development [12]. PSWs also work with adults experiencing mental illness or other behavioral health conditions, such as schizophrenia, bipolar disorder, or depression [4]. These members of society often need, and greatly benefit from, some degree of life-long assistance from a part- or full-time caregiver (paid or unpaid). Assistance with daily living needs can include help with personal hygiene, dressing, medication management, and cooking, as well facilitating participation in social or recreational activities within the community. Some PSWs may also perform job coaching, where they support consumers as they seek jobs and participate in the workforce [4]. The labor of PSWs can be physically hazardous (e.g., assisting with physical transfers or movement) [11], expose them to violence (e.g., hitting or biting) [13], and take an emotional toll (e.g., coping with stress and depression associated with caregiving) [14].

As a result of their unique workplace exposures, PSWs are at an increased risk for a range of outcomes such as injury [15], stress, and burnout [16]. In a recent longitudinal study with caregivers in youth residential institutions, Kind and colleagues [17] found significant associations between exposure to aggression while working and burnout and cortisol levels, respectively. In a review of work-related stress and well-being among care workers in IDD services, Ryan and colleagues [18] drew a thematic categorization of experiences among direct care workers (in residential, community, and hospital settings) that ranged from sources of stress, such as challenging consumer behaviors and role ambiguity, to positive experiences, such as helping to facilitate consumers’ achievement of developmental milestones, appreciation from consumers and families, and positive work motivation.

HCWs share certain job requirements and experiences with PSWs, but care for a population with a different demographic profile and day-to-day needs. For example, like PSWs, HCWs report high satisfaction from close relationships built with consumers and from the meaning and purpose of their work. HCWs are more likely to spend most of their time in the home rather than out in the community, and some research suggests that the majority of their time is spent performing housekeeping tasks [19]. HCWs also typically care for older adults with more typical developmental functioning, although age-related disabilities are often relevant (e.g., dementia). HCWs’ tasks can be physically strenuous, and like PSWs, they are also at an increased risk for physical injury [19]. Tasks such as supporting consumer transfers and assisting with dressing and bathing can result in musculoskeletal pain and injuries. Housekeeping tasks can expose HCWs to prolonged and awkward postures, as well as hazardous cleaning chemicals. For home care aides who assist with medical needs of clients, they are also at-risk for exposure to infectious diseases and injuries from sharps [20].

### 1.2. Experiences of Aggression and Violence Among PSWs and HCWs

Prior research has shown that many types of caregivers, including workers in long-term care, hospital facilities, and in private homes, may experience verbal and physical aggression and violence. For PSWs caring for individuals with IDDs, many of these consumers exhibit challenging behaviors that can cause PSWs physical or emotional harm [8]. Challenging behaviors have been defined as “…culturally abnormal behavior(s) of such intensity, frequency, or duration, that the physical safety of the person or others is likely to be placed in serious jeopardy, or behavior which is likely to limit use of, or result in the person being denied access to, ordinary community facilities” [21] (p. 3). These behaviors can include aggression, self-injury, destructiveness, over activity, inappropriate social or sexual conduct, bizarre mannerisms, and eating inappropriate objects [21]. For this paper, we focus specifically on aggressive challenging behaviors. Consumers with mental health conditions, such as schizophrenia, can also exhibit aggression [22]. Aggression can include threatening harm, yelling and cussing, emotional manipulation, or physical violence including biting, kicking, pushing, or throwing objects [23,24,25]. Specifically relevant to the current project, Hanson and colleagues [9] conducted a study of aggression and violence experienced by Oregon HCWs. They defined three types of aggressive consumer behavior: verbal aggression (e.g., yelling, insulting), workplace aggression (i.e., non-physical threats), and workplace violence (i.e., the occurrence of physical assault). In that study, HCWs’ experiences with these types of challenging and aggressive behaviors were statistically significantly associated with depression, stress, and burnout [9].

### 1.3. Need for Research Comparing Experiences of PSWs and HCWs

Caregivers who work in the community are an important population for research that can inform the design of protective and supportive interventions. Despite the many health and safety hazards of their occupations, these isolated workers often perform their services without the benefit of typical occupational supports and protections available to other more traditional workers, such as safety training [26], environmental safety audits, employer assessment and correction of hazards, as well as co-worker and supervisor support [27].

In Oregon, PSWs and HCWs who provide services to individuals enrolled in publicly funded programs (e.g., funded by Medicaid) are eligible to participate in paid training courses offered by the Oregon Home Care Commission system. Many topics are offered, including managing challenging behaviors. In the Oregon training context, and for other state-based systems, it is important to understand where the experiences and needs of PSWs and HCWs overlap, and where training might need to be tailored to fit PSW’s unique challenges. Challenging and aggressive behaviors may be an important differentiator in training needs. Although both types of caregivers are known to experience and cope with aggression, differences in exposures to such behaviors is poorly defined. It is likely, given the different populations these caregivers serve, that differences in experiences with aggression and worker needs are meaningful. Understanding these potential differences in experiences of PSWs and HCWs, and how they relate to worker outcomes, is critical for informing educational and structural supports to advance their safety, health, and well-being.

To address this research gap, the current study was designed to better understand differences between PSWs and HCWs in their experiences with aggressive challenging behaviors. We also sought to evaluate associations between exposures to aggression and psychological health factors, and describe the PSW sample, including their demographics; work characteristics; illness, injury, and pain experiences; and psychosocial factors. Our project included new data collection with PSWs that was compared with previously published data collected from HCWs [9]. Both studies were conducted in Oregon with workers caring for people who qualified for publicly-funded personal support or home care services. The workers are represented by the Service Employees International Union Local 503. The overall system of care is managed by the Oregon Home Care Commission, which also serves as the employer of record in collective bargaining with the union.

## 2. Materials and Methods

### 2.1. Recruitment, Inclusion, and Data Collection

PSWs were recruited to complete a survey administered via Research Electronic Data Capture (REDCap), a secure electronic survey platform [28,29]. The Oregon Home Care Commission sent a survey link to all PSWs with an active provider number in their system. PSWs who were (a) currently providing services in Oregon to a consumer funded through Medicaid and (b) able to access the Internet (via phone or computer) to complete the online survey were eligible to participate. PSWs who responded and completed the survey (*N* = 248) received a $10 electronic gift card. We did not conduct an a priori power analysis to identify a target sample size because effect sizes were not included in the relevant previous published study with HCWs [9]. Therefore, we allowed our funding level to determine our sample size. The survey was closed after only two days, as available funding was exhausted. All subjects gave their informed consent for inclusion before they participated in the study. The study was conducted in accordance with the Declaration of Helsinki, and all study procedures were approved by the Oregon Health & Science University human subjects Institutional Review Board (IRB00005473).

### 2.2. PSW Characteristics and Work Profile Measures

**Demographics**. Demographic items captured participants’ age, sex, race/ethnicity, and work characteristics.

**Safety Concerns.** In an open-ended question, participants were asked to “list the tasks you complete as a personal support worker where you are most concerned about your physical safety (include tasks that may cause you physical discomfort or pain).”

**Injuries and Illnesses**. Four items assessed experienced injuries, illnesses, and missed workdays due to injury or illness in the past six months. A sample item read, “If you had one or more injuries at work that required you to miss work on following shifts, how many total days did you miss?” with a scale ranging from 0 (no missed days) to 5 (yes, 10 or more). Participants were also asked about workers compensation claims. This item read, “In the last six months, have you filed a worker’s compensation claim?” with a scale ranging from 0 (no, have not filed a claim) to 5 (yes, 5 or more claims).

**Musculoskeletal Pain**. Pain was assessed using an eight-item Nordic style questionnaire adapted from a prior published study [30]. Participants were asked to report whether they experienced musculoskeletal pain in various body regions (neck/shoulders, forearm/wrist, lower back, and lower extremities) in the past 3 months. A sample item read, “During the last 3 months, did you have pain or discomfort in your lower back?” Branching logic was then utilized to assess interference in work or home activities when a participant’s pain was reported in a given region. A sample item reads, “During the last 3 months, how much did pain or discomfort in your lower back interfere with your normal activities?” with a scale of 0 (not at all) to 4 (extremely).

### 2.3. Aggressive Challenging Behaviors and Psychological Health Measures

**Aggressive Challenging Behaviors**. Experiences with aggressive challenging behaviors were assessed with a short version of the Workplace Aggression and Violence Scale [10] as adapted for HCWs in the prior study [9]. Participants were asked to report frequency of problematic or aggressive behaviors experienced “as a personal support worker” in the last 12 months. We included three items that assessed the frequency of verbal aggression (α = 0.83), five items that assessed the frequency of non-sexual workplace aggression (α = 0.82), and nine items that assessed the frequency of non-sexual workplace violence (α = 0.89). Although prior research [10] only distinguished between aggression and violence, we followed Hanson and colleagues’ [9] categorization between verbal aggression (e.g., yelling, insulting), workplace aggression (i.e., threatening behavior), and workplace violence (e.g., pushed, choked). A sample verbal aggression item read, “In the last 12 months of work have you had someone be verbally aggressive with you?” with a scale ranging from 0 (never) to 5 (five times or more). A sample workplace aggression item read, “…had someone try (but fail) to hit you with something?” with a scale ranging from 0 (never) to 5 (five times or more). Sample violence items included, “…been pushed, grabbed, or shoved?” and “…been choked?” rated on a scale of 0 (never) to 5 (five times or more). Branching logic was utilized to capture severity of related injury if applicable. The injury item read, “If you were injured, what was the severity?” rated on a scale of 0 (no injury) to 4 (very serious injury).

**Fatigue**. Fatigue was measured using eight items from the Swedish Occupational Fatigue Inventory (SOFI; α = 0.93) [31]. Participants reported how often they experienced certain feelings at work (e.g., worn out; spent) during the prior month, on a scale ranging from 0 (never) to 4 (very often).

**Stress**. General stress was assessed with the four-item short form version of the Perceived Stress Scale (PSS; α = 0.79) [32]. Examples items included, “Felt confident about your ability to handle your personal problems” (reverse coded) and “Felt that you were unable to control the important things in life”. Items are rated on a scale from 0 (never) to 4 (very often).

**Depression**. Depression was assessed using the five-item Center for Epidemiological Studies Depression Scale (CES-D; α = 0.81) [33]. Example items included, “I felt depressed” and “I had crying spells”. Items were rated on a scale from 0 (rarely or none of the time) to 3 “most of or all of the time” based on the prior 7 days.

## 3. Results

Data were included in analyses for individuals who reported working more than zero hours per week as a PSW and completed all items in the online survey (*N* = 240). All participants in the study were registered as PSWs in the Oregon Home Care Commission registry, but some were also registered as HCWs. A limitation in our dataset is that we did not ask participants to report whether they were also active as a HCW or only registered in the system for personal support work.

### 3.1. Demographics

The mean age of participants was 45.34 years (*SD* = 15.23). About 84% of the participants were white, and 92% were female. PSWs worked an average of 30.38 h per week (*SD* = 20.88). The large standard deviation was influenced by individuals reporting live-in work arrangements with high weekly hours (e.g., 168). Not only did live-in employees (*n* = 25) work significantly more hours per week on average (*M* = 52.28, *SD* = 38.62; *Mdn* = 40.00) than hourly employees (*n* = 215; *M* = 27.84, *SD* = 16.03; *Mdn* = 30.00), *t* (24.97) = 24.45, *p* < 0.001, Levene’s test revealed that the variance across those hours was also much larger for live-in workers (*F* = 52.87, *p* < 0.001). The average time spent in the PSW occupation was 6.6 years (*SD* = 7.51), and PSWs cared for an average of 1.60 (*SD* = 0.9) consumers.

Ages for PSWs’ consumers ranged across the lifespan (ages 5 to over 65), with the most prevalent age groups being 25–44 years (33.1%), 5–17 years (25.7%), and 18–24 years (20.9%). However, we note that only 148 workers (61.7%) reported the age range for their primary consumer. Most PSWs (55.8%, *n* = 134) reported caring for their own children (22.1%, *n* = 53), relatives (21.7%, *n* = 52), or family friends (12.1%, *n* = 29). On average, participants spent much of their time assisting consumers with activities of daily living, social needs, and behavioral needs (Table 1).

### 3.2. Illness, Injury, and Pain Experiences

Many PSWs (41.3%) reported missing one or more workdays due to illness or personal reasons in the past six months. About 27% of PSWs reported experiencing a minor injury in the past six months that did not result in missed work time, and about 4% experienced an injury that did result in lost work time. Four of the 240 workers (1.7%) reported filing a workers’ compensation claim in the prior six months.

Given the sample averaged about 30 h of work per week, we were able to estimate an annual lost time injury rate of 10.7 per 100 full time workers using the formula [34] below:

Annual lost time injury rate per 100 full time workers = number of cases × 200,000/number of employee labor hours worked(1)

- Nine workers experienced 10 total lost time injuries.
- For the reporting period, the sample (*N* = 240) would have worked an estimated 30 h per week over 26 weeks (six months), which is a total of 187,200 estimated hours worked [240 × (30 × 26 weeks)].

Annual lost time injury rate per 100 full time workers = 10 × 200,000/187,200(2)

Annual lost time injury rate per 100 full time workers = 10.7(3)

Self-reported prevalence of pain or discomfort in four body regions over the past three months were: 65.0% for the neck/shoulders; 32.1% for the forearm/wrist; 59.6% for the low back; and 52.1% for lower extremities. Of those reporting pain in these regions, the majority of workers (75–92%, depending on body region) reported the pain interfering with work to some degree (“a little bit” to “extremely”). Overall, 80.4% of the sample reported experiencing some form of pain in the last three months.

### 3.3. Safety Concerns

Responses to the open-ended task-related safety concerns question were coded using recommendations for conventional content analysis [35]. Themes were physical demands (e.g., handling wheelchairs, bathing clients, and moving heavy objects), challenging behaviors (e.g., threats of violence, outbursts, and consumer self-harm), communicable diseases (e.g., exposure to blood, urine, feces, or saliva), stressors (e.g., unpredictability), temperature in client home, long shift hours, driving and transportation, and lack of safety training.

Seventy-two participants (30.1%) reported no task-related safety concerns as a PSW. For the remaining 69.9% of workers, some (*n* = 20) reported more than one primary safety concern. For analyses, responses were coded according to the first issue described. Over half the sample who reported concerns (51.0%; *n* = 122) indicated physical demands as their primary concern, with the next most frequent first reported concern being challenging behaviors (10.5%; *n* = 25). The third most frequent first reported concern was communicable diseases, albeit a much smaller relative proportion (3.3%; *n* = 8). The rest of the first concerns reported added up to 5.0% (*n* = 12).

### 3.4. Aggressive Challenging Behaviors

To facilitate comparisons with a previous sample of HCWs [9], we dichotomized participant experiences with aggression and violence by recoding each item as 0—Never, or 1—One or more times. Next, we compared these prevalence rates to Hanson and colleagues’ [9] reported rates for aggression and violence toward HCWs by computing chi-square difference tests (using the respective sample sizes and estimated counts). As indicated in Table 2, PSWs generally reported higher prevalence rates of experiencing specific types of verbal aggression, workplace aggression, and violence compared to HCWs. In some cases, these rates for specific experiences were up to six times as high as HCWs. In contrast, HCWs shared similar rates of being made to feel guilty, feeling cornered, and being choked. At an aggregated level, prevalence rates for experiencing any verbal aggression (58.3%), workplace aggression (42.9%), and workplace violence (49.2%) were quite high for PSWs.

### 3.5. Fatigue, Depression, and Stress

To further our comparison of PSWs to the HCWs studied by Hanson and colleagues [9], we also conducted similar multiple regression analyses to test the associations between verbal aggression, workplace aggression, and workplace violence, with psychological health outcomes (see Table 3). First, composite variables for verbal aggression, workplace aggression, and workplace violence were created, so that a score of 0 would indicate no experiences of any type of aggression or violence, and 1 would indicate at least one experience of aggression or violence in the past year. Three separate stepwise regression models were computed for each outcome (with fatigue, depression, and stress as dependent variables, respectively). In the first step, covariates were added to the model (education, age, and work tenure). In the next step, verbal aggression, workplace aggression, or workplace violence was added.

None of the covariates were predictive of the health outcomes (*ps* > 0.05). All aggression and violence experiences were positively associated with fatigue, replicating Hanson and colleagues’ [9] results with the similar outcome construct of burnout with HCWs. In contrast to their findings, only workplace aggression was found to be associated with depression, and aggression/violence experiences were generally unrelated to stress. As such, our results indicate that PSWs had a much weaker relationship between aggression/violence and poor psychological health compared to HCWs, while the association with fatigue was consistent across samples (to the degree occupational fatigue and burnout are overlapping constructs).

## 4. Discussion

The current study represents a rare contrast of two types of direct caregivers and their experiences with challenging aggressive behaviors. It also provides a valuable description of a sample of PSWs drawn from a state-wide population.

*Description of the Sample:* The mean age of the PSWs in our study was 45.34 years (*SD* = 15.23), and they were predominantly white (84%) and female (92%). These demographics resembled those observed in our prior prospective intervention trial with Oregon HCWs in the same state-based system [36]. PSWs reported spending most of their time supporting consumers with activities of daily living, social needs, and behavioral needs. This is contrasted with prior findings with HCWs, who appear to spend most of their time performing housekeeping tasks [19]. Most PSWs reported working less than full-time (except for those providing live-in care), which is consistent with HCWs, who, in our prior work, also reported predominantly part time work [36]. Further, it is noteworthy that over half of the PSWs in the study were caregivers for someone they had a close personal relationship with, such as a child, relative, or family friend. This is consistent with Oregon being a state that allows for paid family caregiving [37,38].

Physically strenuous tasks coupled with the potential for challenging behaviors, can impact physical safety and health. Over 40% of the PSWs in our study reported missing work due to illness or personal reasons, and over a quarter reported minor injuries in the past six months. PSWs’ self-reported lost time injury rate (extrapolated to 10.7 per 100 full time workers) was very high relative to the US average (1.17 per 100 full time workers) [39]; however, it was similar to HCWs in our previous work (extrapolated for this paper using a similar formula to 15.3 per 100 full time workers) [36]. While this injury rate is concerning, it should be noted that self-reported injury data will typically be higher than organizationally reported injury data, as injuries are notoriously under-reported in national datasets [34]. About 84% of the sample reported pain in various body regions. PSWs also identified physical demands as their primary safety concern, similar to that of HCWs [19], followed by challenging behaviors, which may not be as prevalent a safety concern with HCWs. The prevalence of physical health symptoms largely mirrors the literature for in-home caregivers [20].

Aggressive challenging behaviors experienced by PSWs and HCWs and association with psychological health: Our comparison of two caregiver groups showed that PSWs and HCWs both experience exposures to aggressive and violent behaviors exhibited by their consumers, or by other individuals in consumers’ homes or communities. Despite many similar levels of exposures, the study showed that PSWs were far more likely than HCWs to experience aggressive challenging behaviors such as having things thrown at them and witnessing self-harm and aggressive behaviors, which has been similarly observed with other workers caring for individuals with IDDs [15,18]. This is a central finding of the current project; PSWs may be at substantial elevated risk for exposure to workplace aggression and violence compared to both HCWs and workers in general and report a substantially elevated rate for lost-work time injuries relative to the general working US population. When individuals with IDDs exhibit physically disruptive, and at times harmful behaviors, the caregivers nearby can get caught in the fray.

In our examination of the role between exposure to challenging aggressive behaviors and psychological health factors, we saw some divergent findings with PSWs relative to HCWs. Similar to HCWs in the Hanson and colleagues study [9], experiencing challenging aggressive behaviors was linked with fatigue. This is not surprising given the amount of cognitive and physical effort workers must invest in anticipating and managing consumers’ challenging behaviors. Exposure to such behaviors at work is known to be associated with fatigue and burnout in other direct care worker populations [17,18,40]. However, in contrast to prior regression analyses with HCWs [9], aggression and violence were more weakly associated with depression, and not significantly associated with stress among PSWs. While our sample of PSWs was substantially smaller than the Hanson and colleagues’ sample of HCWs, and thus possessed lower statistical power to detect effects, standardized regression coefficients observed for depression and stress were relatively small. These findings may indicate that PSWs are less psychologically impacted by challenging behaviors than HCWs and may see such behaviors as less dangerous or stressful. It is possible that PSWs caring for individuals with IDDs, with whom they have close relationships, view aggression as largely unintentional and inherent to their consumers’ struggles with disabilities. These possibilities are supported, in part, by over half the PSWs in our study citing physical demands as their first reported safety concern, and far fewer (10.5%) reporting challenging behaviors or aggression as their first safety concern.

*Possible Modifiers and Future Directions:* Despite elevated experiences with challenging behaviors among PSWs relative to HCWs, we found that these exposures were more weakly associated with depressive symptoms, and not associated with stress. What factors or moderators might explain these null or weaker relationships observed among PSWs relative to those observed with HCWs?

Differences in experienced aggression and psychological outcomes between HCWs and PSWs could point to potential buffering factors that are unique to personal support work, including job role expectations [8,25] and close personal relationships with the populations served [41]. For example, PSWs’ prior knowledge and expectations for working with individuals with IDDs may cause them to expect challenging behaviors, and view management and coping with such behaviors as intrinsic to the job [8]. PSW work can also be profoundly meaningful and fulfilling [8]. Caregiving has been reported to lead to personal growth and an enhanced sense of life purpose [42]. This sense of meaningfulness may be bolstered when individuals care for people with whom they have close personal relationships, especially when caregivers play impactful roles in their consumers’ social and developmental goal accomplishments. Such relationship qualities and meaning in work could buffer the psychological outcomes associated with experiencing aggression. The potential buffering effects of meaningful and close relationships does come with caveats. First, this type of buffering effect, if present, would also be expected in many HCW and consumer relationships. Furthermore, such personal relationships, however gratifying, can be complex. While caregiving-related stressors may be buffered by close personal relationships with the consumer [43], they may also be augmented by worries related to ensuring long-term care and security for their loved ones [44]. For example, caring for a child, family, member, or spouse can give rise to multiple considerations: Does this caregiver-family member role influence the likelihood of injury reporting? Do such relationships cause workers to over-extend themselves and develop severe role ambiguity and conflict? Future research in these areas could be enriched by exploring such questions. 

Individual worker and consumer characteristics, as well as the home setting, may also moderate relationships between exposures to aggression and psychological outcomes. These include workers’ prior training on how to manage challenging behaviors, and their confidence in handling difficult situations. In a review of work-related stress and well-being among care workers in IDD services, Ryan and colleagues [18] identified individual worker self-efficacy for dealing with aggression as a moderator of the relationship between challenging behavior exposures and stress/burnout. In addition, knowledge of how to proceed after experiencing aggression or violence from a consumer could reduce the stress surrounding the incidents. For example, previous qualitative accounts from HCWs reflected worry about violating the Health Insurance Portability and Accountability Act (HIPAA) if they were to report such experiences to their agency supervisors, and in some instances, case managers reported not providing warnings to new workers that such behaviors could be exhibited from a consumer [45]. Age of the consumer and tenure as a PSW may also play roles in the relationship between work exposures and psychological outcomes for PSWs. The prevalence of recipients’ challenging behaviors and PSWs’ learned abilities to manage and cope with such behaviors may both change over time. The average tenure of PSWs in our study was about six years. The house where PSWs work many of their hours may also impact the relationship between aggressive challenging behaviors and psychological health. Research with migrant domestic workers in Hong Kong found that workers in smaller consumer homes tended to experience a higher prevalence of abuse from consumers and employers [46]. These smaller work environments may strengthen the negative psychological outcomes experienced due to one’s continuously reduced ability to remove themselves from the challenging behaviors. This effect may be particularly notable for PSWs working as live-in caregivers (though only a small proportion of our PSW sample were live-in employees). Future longitudinal studies that follow work trajectories of PSWs over time may identify moderating relationships between home environment, work experience, skill level, and coping strategies and psychological outcomes.

Training for PSWs on preventing and managing challenging behaviors is an important area for intervention given their elevated exposures to such behaviors. Such training could prevent injuries, but also buffer against some of the negative psychological outcomes associated with experiencing aggression. Although challenging behaviors are commonly exhibited by individuals with IDDs and mental health diagnoses, they can be prevented, reduced, or managed to minimize harm. Examples of potentially beneficial training topics include the common operant setting events and functions of challenging behaviors (e.g., escape from demands, social attention, and sensory reinforcement) [47], function-based behavior change approaches, pre-planning and prevention tactics for caregiving experiences in the community, de-escalation and challenging behavior management techniques, and resources to utilize after experiencing such challenging behaviors [48,49,50,51,52].

Existing informational fact sheets and training programs on challenging behaviors are available, and could evaluated and/or expanded upon. For example, the University of Minnesota Institute on Community Integration provides several fact sheets explaining approaches to reduce challenging behaviors, such as using distractors, choice-making, and communication alternatives [49]. The Oregon Home Care Commission offers a paid training class on challenging behaviors, but the course has not been formally evaluated for effectiveness. In addition, the Commission offers the COMmunity of Practice and Safety Support (COMPASS) supportive group program as a paid training course series [53]. The original program was developed for HCWs and included a group lesson on communication strategies to work with consumers to reduce hazards in homes. To be more inclusive of PSWs and address their needs, this communication lesson was expanded to address challenging behaviors. The expanded topic, informed by fact sheets from the University of Minnesota [49], defines challenging behaviors, walks the group through example scenarios with a consumer during a community activity, includes a review of the common functions of challenging behaviors and behavioral ABCs (antecedents, behaviors, and consequences), and provides information on de-escalation skills. The program includes interactive activities and goal setting to improve communication and apply behavior management skills. Research on the HCW version of COMPASS, which focused on improving health and reducing injuries, showed that participants reported improved communication and safety behaviors (e.g., ergonomic tool use), and fewer injuries [36]. Future research could examine whether PSWs participants in the program report improved communication and de-escalation skills, and decreases in exposure to aggressive challenging behaviors and their consequences.

*Limitations & Strengths*: Caregivers are able to be simultaneously registered as both a PSW and HCW in Oregon, and participants in this study were not asked to report whether they were also currently actively working as a HCW. Although many questions referred specifically to PSW work, it is possible that responses may have been influenced by dual work experiences. To partially address this limitation, we replicated our results in a sub-sample of participants who reported working full-time as a PSW specifically (i.e., 40 h a week or more), evaluating the consistency of effects for individuals more likely to be working solely or mostly as a PSW. These supplementary tests revealed consistently higher proportions of PSWs experiencing aggressive challenging behaviors in comparison to HCWs (though verbal aggression prevalence was closer with the previous HCW sample). Furthermore, the significant associations (and null effects) between experiencing these behaviors and psychological health remained consistent with our original findings (see Supplementary Tables S1 and S2). Though potential dual status as a HCW/PSW is an important limitation of the study and potential threat to the validity of our findings, these additional analyses, and the fact that our recruitment messaging focused on the PSW role, provide some confidence that PSW exposures and experiences drove our results. Therefore, it is likely the unique work experiences as a PSW played a substantial role in the exposures to aggression and its relationships to their psychological health that we observed.

The study was also potentially limited in sample size, due to the lack of an a priori power analysis and limits of funding. The sample size was smaller relative to the comparison sample from Hanson and colleagues [9]. As such, our results may be biased by lower than desired power—increased probability of a Type II error—in relation to the comparison sample (i.e., the null effects identified may be a function of small sample rather than a true null effect). However, our sample was powered, based on a post hoc consultation of general power tables [54] (p. 158), to detect a medium sized effect for multiple regression analyses with 3–5 independent variables. In addition, unequal sample sizes typically do not bias the results of a chi-square comparison test [55], and we performed recommended statistical corrections for any comparisons that did not meet the necessary chi-square assumptions [56]. Hanson and colleagues’ sample was also recruited in ways that increased the probability that it was representative of the general Oregon HCW population in terms of race and ethnicity. Hanson and colleagues limited their sample to female HCWs and our sample was 92% female. While the overall populations of both types of caregivers is predominantly female, our findings may not be generalizable to male caregivers.

Finally, although we included covariates in our analyses, the cross-sectional design of our study precludes any causal inferences to be made about the impact (or lack thereof) of aggressive challenging behaviors on psychological health. Similarly, we did not measure other forms of aggression that may be experienced by PSWs, including sexual harassment and violence, which occurs in caregiving occupations generally [57] as well as with HCWs [9] and PSWs [8,58] specifically. As such, it is unknown whether the prevalence of these other types of challenging aggressive behaviors are comparable across occupations.

Among its strengths, this is a rare study to compare and contrast work experiences of two closely related, but distinct, caregiving worker groups. Because our sample of PSWs and the comparison HCW group [9] came from the same statewide system for supporting publicly funded caregiving services, it strengthens our ability to make valid comparisons.

## 5. Conclusions

The current study contrasts some work experiences and associated health factors for the two related but different occupational groups—of PSWs and HCWs. By comparing the experiences of both PSWs and HCWs within the same state-based system, we found overlap and differences in work tasks and experiences with challenging aggressive behaviors. Our project in general highlights a greater prevalence of exposure to aggressive behaviors among PSWs relative to HCWs. However, we also observed differences in the psychological impacts of exposure to workplace aggression and violence. Our findings in this focused survey study suggest areas of need for worker training and other interventions, as well as areas for future research and investigation.

## Figures and Tables

**Table 1:** Percent time personal support workers (PSWs) reported spending supporting various consumer needs.

Needs Supported	Mean % Time	SD
Activities of daily living	43.1	28.0
Medical needs	27.6	29.0
Night time needs	10.2	22.1
Social needs	42.1	32.0
Behavioral needs	35.9	32.2
Other needs	13.3	24.2

**Table 2:** Percentage of personal support workers (PSWs) experiencing challenging behaviors relative to home care workers (HCWs).

In the Last 12 Months of Work, Have You…	PSW Yes % (n)	HCW Yes % (n) ^a^	% Difference; Chi-Square Test ^b^
**Verbal Aggression**			
Had someone cry to make you feel guilty	29.2 (70)	29.2 (351)	0%; χ^2^ (1, *N* = 1459) = 0.01, *p* = 0.907
Been yelled at, shouted at, or sworn at	50.0 (120)	41.6 (496)	8.4%; χ^2^ (1, *N* = 1459) = 7.13, *p* = 0.008
Had someone be verbally aggressive with you	42.5 (102)	34.7 (408)	7.8%; χ^2^ (1, *N* = 1459) = 7.19, *p* = 0.007
**Workplace Aggression**			
* Had a door slammed in your face*	29.6 (71)	11.3 (135)	18.3%; χ^2^ (1, *N* = 1459) = 56.65, *p* < 0.001
* Had someone harm themselves in front of you*	14.6 (35)	6.5 (78)	8.1%; χ^2^ (1, *N* = 1459) = 18.80, *p* < 0.001
Been cornered, or placed in a position that was difficult to get out of	19.6 (47)	18.6 (223)	1.0%; χ^2^ (1, *N* = 1459) = 0.22, *p* = 0.638
* Had someone try (but fail) to hit you with something*	20.8 (50)	9.3 (112)	11.5%; χ^2^ (1, *N* = 1459) = 27.55, *p* < 0.001
* Been threatened with a weapon other than a knife or gun*	6.7 (16)	2.2 (26)	4.5%; χ^2^ (1, *N* = 1459) = 14.74, *p* < 0.001
**Workplace Violence**			
* Been choked*	1.3 (3)	0.2 (3)	1.1%; *p* = 0.060 ^d^
* Been spat on or been bumped with unnecessary force ^c^*	30.0 (72)	9.1 (108)	21.9%; χ^2^ (1, *N* = 1459) = 82.86, *p* < 0.05
Been spat on	15.4 (37)	--	--
Been bumped with unnecessary force	27.5 (66)	--	--
* Been slapped, been pushed, grabbed, or shoved, or been bitten, kicked, or hit with a fist ^c^*	31.3 (75)	14.1 (168)	17.2%; χ^2^ (1, *N* = 1459) = 44.08, *p* < 0.05
Been slapped	13.3 (32)	--	--
Been pushed, grabbed, or shoved	27.1 (65)	--	--
Been bitten, kicked, or hit with a fist	19.2 (46)	--	--
* Had someone smash something in your presence or display a loss of control, or had something thrown at you or had someone threaten to throw something at you ^c^*	42.9 (103)	20.8 (248)	22.1%; χ^2^ (1, *N* = 1459) = 55.92, *p* < 0.05
Had someone smash something in your presence or display a loss of control	39.2 (94)	--	--
Had something thrown at you, or had someone threaten to throw something at you	23.3 (56)	--	--
* Been threatened with a knife, or had someone handle a knife near you in a threatening manner*	3.8 (9)	1.6 (19)	2.2%; *p* = 0.036 ^d^

*Note.* PSWs are personal support workers. HCWs are home care workers. Italics represent experience rates for PSWs that were over double that of HCWs in previous studies. ^a^ Data from Hanson et al. [9], female Home Care Workers in Oregon compensated via Medicaid Waiver or having worked as a HCW within 3 months (*N* = 1219). ^b^ Difference tests were computed assuming no missing data from Hanson et al. [9]. ^c^ Hanson et al. [9] combined these items respectively in their frequency rates (i.e., counts reflect participants that experienced at least one of the items). We report the raw frequency rates of both the individual items and the combined items with PSWs, as well as the comparisons of the combined items from HCWs in Hanson et al. ^d^ Due to the small counts, the assumptions of a chi-square difference test were not met. As such, a Fisher’s exact test was conducted.

**Table 3:** Multivariable regressions predicting health outcomes of PSWs from different forms of workplace aggression.

Model	Fatigue ^a^	Depression ^b^	Stress ^b^
Verbal Aggression	5.06 (0.32) *	0.79 (0.12)	0.51 (0.07)
Workplace Aggression	4.88 (0.31) *	0.99 (0.16) *	0.76 (0.11)
Workplace Violence	3.23 (0.21) *	0.65 (0.10)	0.46 (0.07)

*Note.* Values outside parentheses are unstandardized regression coefficients, and values inside parentheses are standardized betas. Verbal Aggression, Workplace Aggression, and Workplace Violence reflect subscales from Hanson et al.’s [9] adaptation of Barling et al.’s [10] Workplace Aggression and Violence Scale. Fatigue reflects the Swedish Occupational Fatigue Inventory [31]. Depression reflects the Center for Epidemiological Studies-Depression Scale [33]. Stress reflects the Perceived Stress Scale-short version [32]. ^a^ Model predicting fatigue controlled for education, age, work tenure, and work hours per week. ^b^ Models predicting depression and stress controlled for education and age. * Regression coefficients were significant (*ps* < 0.05). All other coefficients were non-significant (*ps* > 0.05).

## Supplementary Materials

The following are available online at https://www.mdpi.com/1660-4601/17/15/5486/s1, Table S1: Percentage of full-time PSWs experiencing challenging behaviors relative to HCWs, Table S2: Multivariable regressions predicting health outcomes of full-time PSWs from different forms of workplace aggression.

## References

[1] Cornelis E., Gorus E., Van Schelvergem N., De Vriendt P. (2019). The relationship between basic, instrumental, and advanced activities of daily living and executive functioning in geriatric patients with neurocognitive disorders. Int. J. Geriatr. Psychiatry.

[2] Edemekong P.F., Bomgaars D.L., Levy S.B. (2019). Activities of Daily Living (ADLs).

[3] Home Health Aides and Personal Care Aides. Occupational Outlook Handbook. https://www.bls.gov/ooh/healthcare/home-health-aides-and-personal-care-aides.htm.

[4] Oregon Department of Human Services Oregon Home Care Commission. https://www.oregon.gov/DHS/SENIORS-DISABILITIES/HCC/Pages/index.aspx.

[5] Morris B. (2020). Personal communication.

[6] Ravenswood K., Douglas J. The New Zealand Aged Care Workforce Survey 2016. http://hdl.handle.net/10292/12324.

[7] Sung H.C., Chang S.M., Tsai C.S. (2014). Working in long-term care settings for older people with dementia: Nurses’ aides. J. Clin. Nurs..

[8] Kelly C. (2017). Care and violence through the lens of personal support workers. Int. J. Care Caring.

[9] Hanson G.C., Perrin N.A., Moss H., Laharnar N., Glass N. (2015). Workplace violence against homecare workers and its relationship with workers health outcomes: A cross-sectional study. BMC Public Health.

[10] Barling J., Rogers A., Kelloway K. (2001). Behind closed doors: In-home workers’ experience of sexual harassment and workplace violence. J. Occup. Health Psychol..

[11] Denton M., Zeytinoglu I.U., Davies S., Lian J. (2002). Job stress and job dissatisfaction of home care workers in the context of health care restructuring. Int. J. Health Serv..

[12] Eunice Kennedy Shriver National Institute of Child Health and Human Development. https://www.nichd.nih.gov/health/topics/idds/conditioninfo/default.

[13] Dagnan D., Weston C. (2006). Physical intervention with people with intellectual disabilities: The influence of cognitive and emotional variables. J. App. Res. Intellec. Disab..

[14] Brain C., Kymes S., DiBenedetti D.B., Brevig T., Velligan D.I. (2018). Experiences, attitudes, and perceptions of caregivers of individuals with treatment-resistant schizophrenia: A qualitative study. BMC Psychiatry.

[15] Menckel E., Viitasara E. (2002). Threats and violence in Swedish care and welfare–magnitude of the problem and impact on municipal personnel. Scand J. Caring Sci..

[16] Devereux J.M., Hastings R.P., Noone S.J., Firth A., Totsika V. (2009). Social support and coping as mediators or moderators of the impact of work stressors on burnout in intellectual disability support staff. Res. Dev. Disabil..

[17] Kind N., Eckert A., Steinlin C., Fegert J.M., Schmid M. (2018). Verbal and physical client aggression—A longitudinal analysis of professional caregivers’ psychophysiological stress response and burnout. Psychoneuroendocrinology.

[18] Ryan C., Bergin M., Wells J. (2019). Work-related stress and well-being of direct care workers in intellectual disability services: A scoping review of the literature. Int. J. Dev. Disabil..

[19] Wipfli B., Olson R., Wright R.R., Garrigues L., Lees J. (2012). Characterizing hazards and injuries among home care workers. Home Health Nurse.

[20] Quinn M.M., Markkanen P.K., Galligan C.J., Sama S.R., Kriebel D., Gore R.J., Brouillette N.M., Okyere D., Sun C., Punnett L. (2015). Occupational health of home care aides: Results of the safe home care survey. Occup. Environ. Med..

[21] Emerson E. (2001). Challenging Behaviour: Analysis and Intervention in People with Severe Intellectual Disabilities.

[22] Hodgins S., Cree A., Alderton J., Mak T. (2008). From conduct disorder to severe mental illness: Associations with aggressive behaviour, crime and victimization. Psychol. Med..

[23] Harris P., Humphreys J., Thomson G. (1994). A checklist of challenging behaviour: The development of a survey instrument. Ment. Handicap. Res..

[24] Bowring D.L., Painter J., Hastings R.P. (2019). Prevalence of challenging behaviour in adults with intellectual disabilities, correlates, and association with mental health. Curr. Dev. Disord. Rep..

[25] Campbell C.L. (2016). Incident reporting by health-care workers in noninstitutional care settings. Trauma Violence Abus..

[26] Oregon Home Care Commission (2012). Safety Manual for Home Care Workers and Personal Support. Workers.

[27] Mabry L., Parker K.N., Thompson S.V., Bettencourt K.M., Haque A., Luther Rhoten K., Wright R.R., Hess J.A., Olson R. (2018). Protecting workers in the home care industry: Workers’ experienced job demands, resource gaps, and benefits following a socially supportive intervention. Home Health Care Serv. Q..

[28] Harris P.A., Taylor R., Thielke R., Payne J., Gonzalez N., Conde J.G. (2009). Research electronic data capture (REDCap)—A metadata-driven methodology and workflow process for providing translational research informatics support. J. Biomed. Inform..

[29] Harris P.A., Taylor R., Minor B.L., Elliott V., Fernandez M., O’Neal L., McLeod L., Delacqua G., FDelacqua F., Kirby J. (2019). The REDCap consortium: Building an international community of software partners. J. Biomed. Inform..

[30] Dennerlein J.T., Hopcia K., Sembajwe G., Kenwood C., Stoddard A.M., Tveito T.H., Hashimoto D.M., Sorensen G. (2012). Ergonomic practices within patient care units are associated with musculoskeletal pain and limitations. Am. J. Ind. Med..

[31] Åhsberg E. (2000). Dimensions of fatigue in different working populations. Scand J. Psychol..

[32] Cohen S., Kamarck T., Mermelstein R. (1983). A global measure of perceived stress. J. Health Soc. Behav..

[33] Bohannon R.W., Maljanian R., Goethe J. (2003). Screening for depression in clinical practice: Reliability and validity of a five-item subset of the CES-Depression. Percept Mot. Ski..

[34] Galizzi M., Miesmaa P., Punnett L., Slatin C. (2010). Phase in Healthcare Research Team. Injured workers’ underreporting in the health care industry: An analysis using quantitative, qualitative, and observational data. Ind. Relat..

[35] Hsieh H.F., Shannon S.E. (2005). Three approaches to qualitative content analysis. Qual. Health Res..

[36] Olson R., Thompson S.V., Elliot D.L., Hess J.A., Rhoten K.L., Parker K.N., Wright R.R., Wipfli B., Bettencourt K.M., Buckmaster A. (2016). Safety and health support for home care workers: The COMPASS randomized controlled trial. Am. J. Public Health.

[37] Niesz H., Martino P. States that Allow Family Members to Act as Personal Care Assistants. https://www.cga.ct.gov/2003/rpt/2003-R-0040.htm#:~:text=Twelve%20states%20.

[38] USA Government Caregiver support. https://www.usa.gov/disability-caregiver#item-213957.

[39] Nonfatal Occupational Injuries and Illnesses Requiring Days away from Work. https://www.bls.gov/news.release/archives/osh2_11092010.pdf.

[40] Gray-Stanley J.A., Muramatsu N. (2011). Work stress, burnout, and social and personal resources among direct care workers. Res. Dev. Disabil..

[41] Karlsson N.D., Markkanen P.K., Kriebel D., Galligan C.J., Quinn M.M. (2020). “That’s not my job”: A mixed methods study of challenging client behaviors, boundaries, and home care aide occupational safety and health. Am. J. Ind. Med..

[42] Haley W.E., Perkins E.A. (2004). Current status and future directions in family caregiving and aging people with intellectual disabilities. J. Pol. Pract. Intellect Disabil..

[43] Heller T., Arnold C.K., van Heumen L., McBride E.L., Factor A. (2012). Self-directed support: Impact of hiring practices on adults with intellectual and developmental disabilities and families. Am. J. Intellect Dev. Disabil..

[44] Bigby C., Whiteside M., Douglas J. (2019). Providing support for decision making to adults with intellectual disability: Perspectives of family members and workers in disability support services. J. Intellect Dev. Disabil..

[45] Nakaishi L., Moss H., Weinstein M., Perrin N., Rose L., Anger W.K., Hanson G.C., Christian M., Glass N. (2013). Exploring workplace violence among home care workers in a consumer-driven home health care program. Workplace Health Saf..

[46] Lai Y., Fong E. (2020). Work-related aggression in home-based working environment: Experiences of migrant domestic workers in Hong Kong. Am. Behav. Sci..

[47] Beavers G.A., Iwata B.A., Lerman D.C. (2013). Thirty years of research on the functional analysis of problem behavior. J. Appl. Behav. Anal..

[48] Services Alternatives Inc. Training Institute De-Escalate Anyone, Anywhere, Anytime: Unplug the Power Struggle with Principle Based De-Escalation. https://rightresponse.org/de-escalation-skills.

[49] Institute on Community Integration LEND Fact Sheet Series: Challenging Behaviors. https://lend.umn.edu/sites/lend.umn.edu/files/2018-08/FS_Challenging_Behaviors-1.pdf.

[50] Reichle J., Davis C., Neilson S., Duran L. (2009). Addressing the Needs of Young Children Who Engage in Challenging Behaviors.

[51] Duran L., Watson C., Gatti S.N. (2009). Recognizing and Coping with Signs of Distress in Young Children.

[52] Iroku-Malize T., Grissom M. (2018). The agitated patient: Steps to take, how to stay safe. J. Fam. Pract..

[53] Olson R., Hess J.A., Parker K.N., Thompson S.V., Rameshbabu A., Luther Rhoten K., Marino M. (2018). From research-to-practice: An adaptation and dissemination of the COMPASS program for home care workers. Int. J. Environ. Res. Public Health.

[54] Cohen J. (1992). A power primer. Psychol. Bull..

[55] Kang S.-H., Ahn C.W. (2008). Tests for the homogeneity of two binomial proportions in extremely unbalanced 2 × 2 contingency tables. Stat. Med..

[56] McHugh M.L. (2013). The chi-square test of independence. Biochem. Med..

[57] Nielsen M.B.D., Kjær S., Aldrich P.T., Madsen I.E.H., Friborg M.K., Rugulies R., Folker A.P. (2017). Sexual harassment in care work—Dilemmas and consequences: A qualitative investigation. Int. J. Nurs. Stud..

[58] Denton M., Zeytinoglu I.U., Brookman C., Davies S., Boucher P. (2018). Personal support workers’ perception of safety in a changing world of work. Saf. Health.

