# Supplementary material for: Challenging Aggressive Behaviors Experienced by Personal Support Workers in Comparison to Home Care Workers: Relationships between Caregiver Experiences and Psychological Health
Source: Int J Environ Res Public Health. 2020 Jul 29;17(15):5486. doi: 10.3390/ijerph17155486 (PMC7432386; doi:10.3390/ijerph17155486)
Supplement: Supplementary file 1 [file ijerph-17-05486-s001.pdf]

Supplementary Table 1. Percentage of full-time PSWs experiencing challenging behaviors relative to HCWs

In the last 12 months of work, have you…	PSW Yes% (n) [a]	HCW Yes% (n) [b]	% Difference; Fisher's Exact Test [cd]
**Verbal Aggression**			
Had someone cry to make you feel guilty	33.8 (27)	29.2 (351)	4.6%; *p*=.373
Been yelled at, shouted at, or sworn at	48.8 (39)	41.6 (496)	7.2%; *p*=.161
Had someone be verbally aggressive with you	38.8 (31)	34.7 (408)	4.1%; *p*=.332
**Workplace Aggression**			
Had a door slammed in your face	32.5 (26)	11.3 (135)	21.2%; *p*<.001
Had someone harm themselves in front of you	21.3 (17)	6.5 (78)	14.8%; *p*<.001
Been cornered, or placed in a position that was difficult to get out of	27.5 (22)	18.6 (223)	8.9%; *p*=.054
Had someone try (but fail) to hit you with something	28.7 (23)	9.3 (112)	19.4%; *p*<.001
Been threatened with a weapon other than a knife or gun	13.8 (11)	2.2 (26)	11.6%; *p*<.001
**Workplace Violence**			
Been choked	3.8 (3)	0.2 (3)	3.6%; *p*=.004
Been spat on or been bumped with unnecessary force [e]	41.3 (33)	9.1 (108)	21.9%; *p*<.001
Been spat on	25.0 (20)	--	--
Been bumped with unnecessary force	40.0 (32)	--	--
Been slapped, been pushed, grabbed, or shoved, or been bitten, kicked, or hit with a fist [e]	40.0 (32)	14.1 (168)	25.9%; *p*<.001
Been slapped	20.0 (16)	--	--
Been pushed, grabbed, or shoved	33.8 (27)	--	--
Been bitten, kicked, or hit with a fist	31.3 (25)	--	--

In the last 12 months of work, have you…	PSW Yes% (n) [a]	HCW Yes% (n) [b]	% Difference; Fisher's Exact Test [cd]
Had someone smash something in your presence or display a loss of control, or had something thrown at you or had someone threaten to throw something at you [e]	48.8 (39)	20.8 (248)	28.0%; $p<.001$
Had someone smash something in your presence or display a loss of control	47.5 (38)	--	--
Had something thrown at you, or had someone threaten to throw something at you	27.5 (22)	--	--
Been threatened with a knife, or had someone handle a knife near you in a threatening manner	3.8 (3)	1.6 (19)	2.2%; $p=.149$

Note. PSWs are personal support workers, and HCWs are home care workers. Italics represent experience rates for PSWs that were over double that of HCWs from Hanson et al. [9]. [a] Data from a subsample of PSWs working full-time (40 hours or more) as a PSW (n = 80). [b] Data from Hanson et al. [9], female Home Care Workers in Oregon compensated via Medicaid Waiver or having worked as an HCW within 3 months (N = 1219). [c] Difference tests were computed assuming no missing data from Hanson et al. [9]. [d] Due to the substantially smaller sample size of PSWs, a Fisher's exact test was conducted. [e] Hanson et al. [9] combined these items respectively in their frequency rates (i.e., counts reflect participants that experienced at least one of the items). We report the raw frequency rates of both the individual items and the combined items with PSWs, as well as the comparisons of the combined items from HCWs in Hanson et al.

Supplementary Table 2. Multivariable regressions predicting health outcomes of full-time PSWs from different forms of workplace aggression

Model	Fatigue [a]	Depression [b]	Stress [b]
Verbal Aggression	6.54 (0.40)*	0.48 (0.07)	0.20 (0.03)
Workplace Aggression	5.47 (0.33)*	1.19 (0.18)	0.75 (0.11)
Workplace Violence	6.98 (0.43)*	0.91 (0.14)	0.97 (0.14)

Note. Values outside parentheses are unstandardized regression coefficients, and values inside parentheses are standardized betas. Verbal Aggression, Workplace Aggression, and Workplace Violence reflect subscales from Hanson et al.'s [9] adaptation of Barling et al.'s [10] Workplace Aggression and Violence Scale. Fatigue reflects the Swedish Occupational Fatigue Inventory [31]. Depression reflects the Center for Epidemiological Studies-Depression Scale [33]. Stress reflects the Perceived Stress Scale-short version [32]. [a] Models predicting fatigue controlled for education, age, work tenure and work hours per week. [b] Models predicting depression and stress controlled for education and age.

*Regression coefficients were significant (*ps*<.05). All other coefficients were non-significant (*ps*>.05).
